# Recent advances in the regulatory and non-coding RNA biology of osteogenic differentiation: biological functions and significance for bone healing

**DOI:** 10.3389/fcell.2024.1483843

**Published:** 2025-01-06

**Authors:** Samaris Aranguren, Hisae Cole, Lauren Jeannette Dargan, Madison Sarlo, Sumin Choi, Isha Satapathy, Jaira Ferreira de Vasconcellos

**Affiliations:** Department of Biology, James Madison University, Harrisonburg, VA, United States

**Keywords:** non-coding RNAs, regulatory RNAs, osteogenic differentiation, bone fracture, bone healing, circular RNAs, small nucleolar RNAs, PIWI-interacting RNAs

## Abstract

Injuries associated with contemporary life, such as automobile crashes and sports injuries, can lead to large numbers of traumatic neuromuscular injuries that are intimately associated with bone fractures. Regulatory and non-coding RNAs play essential roles in multiple cellular processes, including osteogenic differentiation and bone healing. In this review, we discuss the most recent advances in our understanding of the regulatory and non-coding RNA biology of osteogenic differentiation in stem, stromal and progenitor cells. We focused on circular RNAs, small nucleolar RNAs and PIWI-interacting RNAs and comprehensively summarized their biological functions as well as discussed their significance for bone healing and tissue regeneration.

## 1 Introduction

Globally, the remarkable number of 178 million new bone fractures have been reported in the year of 2019 (Wu et al., 2021). Since the year of 1990, an increase of 70.1% in the absolute number of acute or long-term symptoms of a fracture as well as an increase of 65.3% in the absolute number of years lived with a disability as a result of a fracture were observed (Wu et al., 2021). In the United States, 5.6 million fractures per year have been reported and affect nearly 2% of the population (Wang et al., 2017). Based on the estimated growth of the elderly population, age-related fractures alone, which often are fragility fractures, are estimated to account for over 3 million fractures per year by 2025 (Burge et al., 2007). The most commonly reported fracture sites are the distal radius, proximal femur, ankle, proximal humerus and metacarpal (Bergh et al., 2020). Overall, bone fractures are considered a global public health issue and the cause of a significant societal burden, including a noteworthy economic overload (due to events such as hospitalization, surgery, days away from work, among others) and a negative impact on the quality of life of the individuals affected.

The biology of bone healing is a complex process; fractures heal through two distinct mechanisms: (i) primary (or direct) healing, where mesenchymal stem cells transition directly into osteoblasts (a process known as intramembranous ossification) and (ii) secondary (or indirect) healing, where cartilage is formed as an intermediate step prior to osteoblastic-induced bone formation (a process known as endochondral ossification). In addition, multiple cell types (such as inflammatory cells, mesenchymal stem/stromal/progenitor cells, endothelial cells, osteoblasts, chondrocytes and osteoclasts), as well as different molecular players (for example, cytokines, growth factors and transcription factors) play a role in the different cellular and molecular mechanisms underlying bone and fracture healing (Bahney et al., 2019). Our current understanding of bone and fracture healing divides the process into the following phases: (i) inflammatory, (ii) fibrovascular, (iii) bone formation and (iv) callus remodeling (Bahney et al., 2019). Finally, recent and significant advances in the field of RNA biology have highlighted the critical role that regulatory and non-coding RNAs play in the regulation and the differentiation of stem, stromal and progenitor cells into the osteogenic lineage (Hassan et al., 2015; Huo et al., 2018; Li, 2018; Ju et al., 2019; Guo B. et al., 2021; Lan et al., 2024), with direct consequences in bone healing after a traumatic event.

In this review, we focused on recent advances related to the role of three critical groups of less abundant regulatory and non-coding RNAs: circular RNAs (circRNAs), small nucleolar RNAs (snoRNAs) and PIWI-interacting RNAs (piRNAs) in the context of osteogenic differentiation of mesenchymal stem, stromal and progenitor cells and bone healing. Despite their importance, we chose not to focus on the more abundant groups of regulatory and non-coding RNAs - microRNAs and long non-coding RNAs - during osteogenic differentiation because they have been previously comprehensively reviewed (Peng et al., 2016; Hodges et al., 2017; Li, 2018; Valenti et al., 2018; Yang et al., 2018; Wang et al., 2019; Sun et al., 2020; Wang et al., 2020; Guo Z. et al., 2021; Lanzillotti et al., 2021; Zhou et al., 2021; An et al., 2023). Finally, we discussed the significance of circRNAs, snoRNAs and piRNAs for translational applications in bone fracture and healing.

## 2 Osteogenic lineage commitment, osteogenic differentiation and bone healing

Injuries associated with modern life may lead to traumatic neuromuscular injuries that are closely associated with bone fractures. Post-traumatic bone fractures may be triggered directly or indirectly by external impact and are most likely the result of fragile bone structures following a traumatic event (Lai et al., 2022). Bone fractures are a significant challenge in the orthopedics field and have very important clinical and economic consequences. Treatment strategies will vary and depend on the complexity of the fracture and may include immobilization, a closed reduction procedure (non-surgical) or an open reduction procedure (surgical) with internal or external fixation, which provides the stability to maintain an appropriate alignment and accelerates bone healing, with the ultimate goal of restoring bone function (Dingle et al., 2021; Lai et al., 2022). However, despite the available treatment options and the recent advances in orthopedics and orthopedic surgery fields, patients with bone fractures may suffer from treatment strategies that fail and/or are insufficient to heal the fractures, which may result in a state of bone deficiency that, in some cases, may need bone replacement (Meesuk et al., 2022). As such, a better understanding of the molecular mechanisms underlying bone formation as well as bone healing processes are needed and an important gap in knowledge for the successful treatment of bone fractures.

**FIGURE 1:**
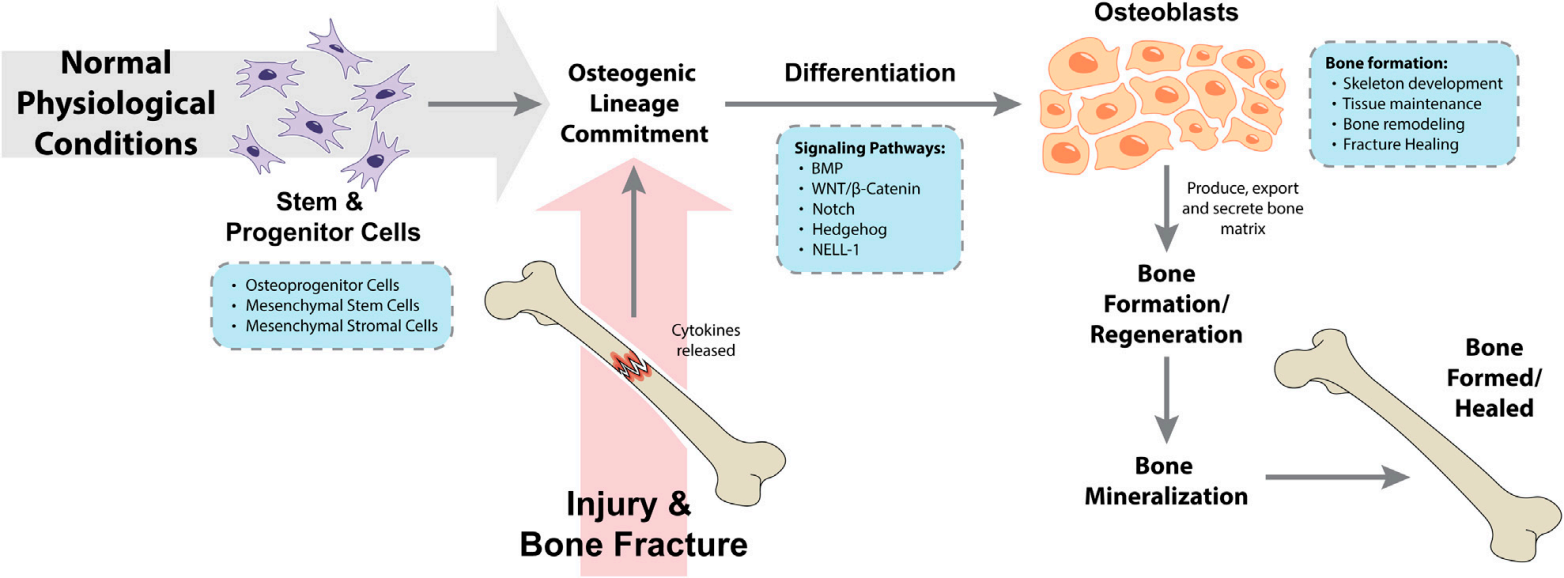
Processes and pathways involved in osteogenic lineage commitment and osteogenic differentiation during normal physiological conditions and following a traumatic injury. BMP, bone morphogenetic protein; NELL-1, neural epidermal growth factor-like 1 protein.

Osteogenic differentiation is the process where osteoprogenitor cells as well as mesenchymal stem and/or stromal cells differentiate to form osteoblasts, which under normal physiological conditions are the cells responsible for bone formation during skeleton development, throughout life (tissue maintenance and bone remodeling) and during post-traumatic fracture healing (Valenti et al., 2016; Thomas and Jaganathan, 2022). Prior to the osteogenic differentiation itself, mesenchymal stem and/or stromal cells must commit to differentiate into the osteogenic lineage, and not – for example – differentiate towards the adipogenic and chondrogenic lineages. This complex and regulated process is known as mesenchymal stem and/or stromal cells osteogenic lineage commitment (Montecino et al., 2021). Following injury and bone fracture, cytokines are released by the injured bone, leading to the mesenchymal stem and/or stromal cells differentiation into progenitor cells, pre-osteoblast cells and osteoblasts. As a result, osteoblasts will produce, export and secrete bone matrix as well as aid in the tissue repair process in the cellular microenvironment, which altogether will trigger bone regeneration (Zhou et al., 2021). Finally, bone mineralization consists in the incorporation of minerals (such as calcium and phosphorus) by osteoblast phosphatases to the matrix at sites of newly formed bone, leading to a bone structure with more physical density (Land and Schoenau, 2008; Valenti et al., 2016). An overview of these processes is summarized in Figure 1. The role of osteoblast cells in normal bone physiology and during bone metastasis has been reviewed elsewhere (Ottewell, 2016; Valenti et al., 2016).

Mesenchymal stem and/or stromal cells isolated from multiple tissues [such as, but not limited to, bone marrow, dental pulp, adipose tissue and umbilical cord blood (Rebelatto et al., 2008; Shima et al., 2015; Kawashima et al., 2017; Si et al., 2019)] as well as mesenchymal progenitor cells (MPCs) isolated from traumatized muscle following high and low energy trauma (Nesti et al., 2008; Dingle et al., 2021) have shown multipotent differentiation capacity, including the ability to differentiate into the osteogenic lineage. However, depending on the tissue of origin and replicative senescence, variations in mesenchymal stem and/or stromal cells’ biological properties and their ability to differentiate into the osteogenic lineage have been observed (Grotheer et al., 2021).

Human mesenchymal stem and/or stromal cells osteogenic lineage commitment and osteogenic differentiation are tightly regulated processes occurring in multiple steps and regulated by mechanical signals and several different signaling pathways. The signaling networks regulating osteogenesis have been previously reviewed (Valenti et al., 2016; Montecino et al., 2021; Thomas and Jaganathan, 2022). Briefly, osteogenic differentiation is known to be modulated by bone morphogenetic proteins (BMPs) signaling, WNT/β-catenin signaling, Notch signaling, Hedgehog signaling, the neural epidermal growth factor-like 1 protein (NELL-1) signaling and multiple transcription factors (Thomas and Jaganathan, 2022). BMP signaling is a major regulator of osteogenic differentiation and has been shown to synergistically interact with WNT/β-catenin, Hedgehog and NELL-1 signaling to promote mesenchymal stem and/or stromal cells' osteogenic lineage commitment and osteogenic differentiation (Thomas and Jaganathan, 2022). BMPs play a role in osteogenic differentiation by activating the transforming growth factor β/SMAD kinase receptors signaling pathway and the runt-related transcription factor 2 (RUNX2) transcription factor, which can also be activated by the WNT/β-catenin signaling pathway (Valenti et al., 2016). Additional genes and proteins required and/or involved in osteogenic differentiation and bone formation include the Sp7 transcription factor (OSTERIX), secreted protein acidic and cysteine rich (SPARC; also known as osteonectin), secreted phosphoprotein 1 (SPP1; also known as osteopontin) and collagen type I alpha 1 chain (COL1A1). *Runx2* and *Osterix* transcription factors are essential for mesenchymal stem cells (MSCs) osteogenic lineage commitment and, as a result, for osteogenic differentiation (Montecino et al., 2021). More recently, regulatory and non-coding RNAs have also been reported as critical players in the processes of osteogenic differentiation, osteoblastic lineage commitment and bone formation; the more abundant microRNAs and long non-coding RNAs have been extensively studied and reviewed (Peng et al., 2016; Hodges et al., 2017; Li, 2018; Valenti et al., 2018; Yang et al., 2018; Wang et al., 2019; Sun et al., 2020; Wang et al., 2020; Guo B. et al., 2021; Lanzillotti et al., 2021; Zhou et al., 2021; An et al., 2023), while the less abundant, and more recently investigated, circRNAs, snoRNAs and piRNAs are reviewed here. Altogether, these molecular players highlight the complexity of bone healing and the transcriptional and post-transcriptional regulation of the distinct, but interconnected processes of mesenchymal stem and/or stromal cells osteogenic lineage commitment, osteogenic differentiation and bone formation.

## 3 Regulatory and non-coding RNAs biological functions in stem, stromal and progenitor cells during osteogenic differentiation

### 3.1 Circular RNAs and osteogenic differentiation

CircRNAs are single-stranded RNAs that form a continuous and covalently closed loop. CircRNAs are generated by non-canonical messenger RNA (mRNA) back-splicing events where the 3′terminal end of the RNA is directly linked to the 5′terminal end during the splicing of the precursor mRNA and, as a result, form a continuous and circular structure (Lei et al., 2020; Yang et al., 2021). CircRNAs are highly conserved across evolution, very stable due to their circular covalently closed structure (and, as a result, more resistant to the degradation of RNA exonucleases), and expressed in a tissue-specific, subcellular location- and developmental stage-specific manner (Salzman et al., 2013; He et al., 2021; Yang et al., 2021). The biogenesis and degradation of circRNAs have been reviewed elsewhere (He et al., 2021; Yang et al., 2021; He and Zhu, 2023). Of interest, although initially described and generally considered as non-coding RNAs, some endogenous circRNAs have been shown to be associated with translating ribosomes and, as such, are considered protein-coding circRNAs (Legnini et al., 2017; Pamudurti et al., 2017; Lei et al., 2020; Yang et al., 2021). CircRNAs play important roles in a diverse range of cellular processes, such as acting as microRNAs sponges to modulate (upregulate) the expression of their target mRNA (Hansen et al., 2013), regulating transcription and translation (Li et al., 2015; Wu et al., 2019) and promoting/facilitating the interactions between proteins (Wu et al., 2019; Fang et al., 2018). Moreover, dysregulation of circRNAs is associated with the pathogenesis of various diseases, including cancers as well as neurological and cardiovascular diseases and the detection of circRNAs as biomarkers and/or the manipulation of circRNAs as therapeutic targets have a promising potential for future clinical interventions (Zhang et al., 2018; Santer et al., 2019; He et al., 2021; Liu A. et al., 2022; He and Zhu, 2023).

In the process of bone healing, some circRNAs play a role in the molecular mechanisms regulating stem, stomal and progenitor cells undergoing osteogenic differentiation through an orchestrated direct or indirect regulation of osteogenic-related genes. A study using human primary mesenchymal stromal cells induced to osteogenic or chondrogenic differentiation for 7 days identified the differential expression of circRNAs, microRNAs and piRNAs after commitment towards the osteogenic or chondrogenic lineage (Della Bella et al., 2020). The RNA sequencing analysis identified 21 upregulated and 21 downregulated circRNAs in osteogenic induced cells compared to the control (Della Bella et al., 2020). The top 10 upregulated circRNAs based on the fold change (fold change range from 5.4 to 2.0) were: *hsa_circRNA_104101*, *hsa_circRNA_002161*, *hsa_circRNA_076155*, *hsa_circRNA_022382*, *hsa_circRNA_100833*, *hsa_circRNA_406763*, *hsa_circRNA_100834*, *hsa_circRNA_103249*, *hsa_circRNA_406308* and *hsa_circRNA_405468* (Della Bella et al., 2020). The top 10 downregulated circRNAs based on the fold change (fold change range from 0.6 to 0.4) were: *hsa_circRNA_100512*, *hsa_circRNA_100511*, *hsa_circRNA_100835*, *hsa_circRNA_104981*, *hsa_circRNA_002415*, *hsa_circRNA_103729*, *hsa_circRNA_100509*, *hsa_circRNA_100510*, *hsa_circRNA_103415* and *hsa_circRNA_102854* (see original paper for the complete list of upregulated and downregulated circRNAs and chondrogenic modulated regulatory RNAs; Della Bella et al., 2020). Exploratory studies such as the Della Bella et al. (2020) are very useful to generate a large amount of data that is now publicly available to the scientific community. Additional microarray and/or RNA sequencing studies that have investigated the expression profiles of circRNAs during osteogenic differentiation in different sources of stem cells or MC3T3-E1 cells have been previously reported (Gu et al., 2017; Zheng et al., 2017; Long et al., 2018; Zhang et al., 2019; Qian et al., 2017).

Two recent studies identified the circular RNA *AFF4* (*circ_AFF4*) as playing a role in osteogenic differentiation through the FNDC5/Irisin axis (Liu C., et al., 2021; Liu A. et al., 2022). Liu C. et al. (2021) and Liu A. et al. (2022) reported that *circ_AFF4* was progressively and significantly upregulated during the osteogenic differentiation (up to 14 days) of bone marrow-derived mesenchymal stem cells (BM-MSCs) and that knockdown of *circ_AFF4* inhibited osteogenic differentiation of BM-MSCs. Mechanistically, Liu C. et al. (2021) identified *miR-135a-5p* as the downstream target of *circ_AFF4* that upregulates the FNDC5/Irisin axis to regulate osteogenic differentiation. Finally, bone formation was inhibited *in vivo* following the knockdown of *circ_AFF4* in BM-MSCs cultured on scaffold material and implanted in mice (Liu C. et al., 2021). Moreover, Liu A. et al. (2022) identified that *circ_AFF4* interacts with the RNA binding protein insulin like growth factor 2 mRNA binding protein 3 (IGF2BP3) to stabilize *FNDC5* transcripts. IGF2BP3 has also been recently found to form an RNA-protein complex with *circEIF4B* and, as a result, stabilize *ITGA5* transcripts and promote BM-MSC osteogenic differentiation (Wu et al., 2024). Wu et al. (2024) found *circEIF4B* to be upregulated in BM-MSCs stimulated with phytic acid under a high-glucose microenvironment, and overexpression of *circEIF4B* promoted osteogenic differentiation in these culture conditions. Mechanistically, *circEIF4B* was found to sponge *miR-186-5p*, allowing FOXO1 levels to increase and favor phytic acid-induced high-glucose mediated osteogenic differentiation of BM-MSCs (Wu et al., 2024). Of note, we have also previously reported a role for the *IGF2BP1-3* genes during human *in vitro* osteogenic differentiation in BM-MSCs (Melton et al., 2023).

Another circRNA recently reported to regulate osteogenic differentiation of mouse adipose-derived mesenchymal stem cells (mAD-MSCs) was *mmu-circRNA-23525* (Guo Z. et al., 2021). This study is a follow-up from a previous large expression profile from the same research group that identified a differential expression signature for circRNAs at different stages of osteogenic differentiation in mAD-MSCs (Long et al., 2018). Guo Z. et al. (2021) confirmed that *mmu-circRNA-23525* was significantly upregulated on osteogenic differentiation days 3, 7 and 14, and that overexpression of *circRNA-23525* promoted osteogenic differentiation evidenced by increased expression of the osteogenic markers alkaline phosphatase (*Alp*), osteocalcin (*Ocn*) and runt-related transcription factor 2 (*Runx2*) 3 days after osteogenic induction, increased alkaline phosphatase activity 7 days after induction as well as increased calcium deposition 14 days after induction (Guo Z. et al., 2021). Mechanistically, they found that *circRNA-23525* acts as a sponge for *miR-30a-3p* and regulates the expression of the osteogenic transcription factor *Runx2* in mAD-MSCs (Guo Z. et al., 2021).

Human and rat adipose-derived stem cells were used in additional studies investigating circRNAs during osteogenic differentiation. The circRNA *hsa_circ_0001320* (also named, *circFOXP1*) was significantly downregulated in the bone tissues of osteoporotic patients compared to non-osteoporotic patients, while it was upregulated during the osteogenic differentiation (days 3, 7 and 14 compared to day 0) of human adipose-derived stem cells (hAD-MSCs) (Shen et al., 2020). Moreover, over-expression of *circFOXP1* enhanced the osteogenic differentiation of hAD-MSCs assessed by alkaline phosphatase staining and activity on osteogenic differentiation day 7 and calcium deposits accumulation on osteogenic differentiation day 14 (Shen et al., 2020). Mechanistically, *circFOXP1* acted as a sponge for *miR-33a-5p* to regulate the expression of *FOXP1* and promote hAD-MSCs osteogenic differentiation (Shen et al., 2020). *CircFOXP1* was also shown to regulate hAD-MSCs osteogenesis *in vivo* through *miR-33a-5p* and *FOXP1* using collagen-based hydrogels that were implanted in the dorsal subcutaneous space of a nude mouse model (Shen et al., 2020). The *circRNA-0879* (also named, *circRNA-vgll3*) was upregulated during bone morphogenetic protein 2 (BMP2)-induced osteogenic differentiation (days 2, 7 and 14 compared to day 0) of adipose-derived stem cells isolated from Sprague Dawley rats (rAD-MSCs) (Zhang et al., 2021). Overexpression of *circRNA-vgll3* positively regulated BMP2-induced osteogenic differentiation of rAD-MSCs as evidenced by the significantly increased expression levels of osteogenic markers [*Runx2*, *Ocn*, osterix (*OSX*), collagen1 a1 (*Col1a1*), osteopontin (*Opn*) and bone sialoprotein (*Bsp*)] compared to the transfection control as well as significantly increased levels of alkaline phosphatase activity and calcium deposition in the *circRNA-vgll3* overexpression compared to the transfection control (Zhang et al., 2021). The reverse results were observed upon the knockdown of *circRNA-vgll3* with an inhibitor compared to the control (Zhang et al., 2021). Mechanistically, it was shown that *circRNA-vgll3* binds to *miR-326-5p* and regulates the osteogenic differentiation of rAD-MSCs by targeting *Itga5* transcripts (Zhang et al., 2021). Additional experiments were performed to investigate the potential for *circRNA-vgll3* to heal injured bone *in vivo* using a critical-sized bone defect model in rat skulls and demonstrated that *circRNA-vgll3*-engineered rAD-MSCs positively regulated bone regeneration and new bone formation *in vivo* (Zhang et al., 2021).

Additional studies found a circRNA expression signature during the osteogenic differentiation (days 3, 7 and 14 compared to day 0) of periodontal ligament stem cells (PDLSCs) (Zheng et al., 2017) as well as a role for the selected circRNA antisense to the cerebellar degeneration-related protein 1 transcript (*CDR1as*) in the osteogenic differentiation of human primary PDLSCs (Li et al., 2018). *CDR1as* was significantly upregulated following 3, 7 and 14 days of osteogenic differentiation of human primary PDLSCs compared to day 0, and knockdown of *CDR1as* inhibited osteogenic differentiation as evidenced by decreased levels of alkaline phosphatase activity (after 7 days of osteogenic differentiation) and calcium deposition (after 14 days of osteogenic differentiation) in the *CDR1as*-knockdown compared to the transfection control (Li et al., 2018). Mechanistically, *CDR1as* was shown to sponge *miR-7* and regulate the growth differentiation factor 5 (*GDF5*) (Li et al., 2018). Additionally, the *CDR1as*-*miR-7*-*GDF5* axis induced osteogenic differentiation of human primary PDLSCs, at least in part, by activating the phosphorylation of Smad1/5/8 and p38 mitogen-activated protein kinase (MAPK) (Li et al., 2018). Lastly, human primary PDLSCs seeded on scaffold materials were implanted in the calvarial defect area of nude mice for 8-weeks and demonstrated that knockdown of *CDR1as* inhibited the formation of new bone *in vivo* as shown by micro-CT and histological analyses compared to the control group (Li et al., 2018).

Additional circRNAs that have been found to be associated with the osteogenic differentiation of stem cells are: *hsa_circRNA_33287*, *hsa_circ_0003489* (*circCDK8*), *hsa_circ_0113689* (*circ-DAB1*) and *circLPAR1*. The circRNA *hsa_circRNA_33287* was discovered through the microarray analysis of primary human maxillary sinus membrane stem cells (MSMSCs) cultured in BMP2-induced osteogenic differentiation conditions compared to the control (Peng et al., 2019). The microarray analysis found a total of 32 upregulated circRNAs and 18 downregulated circRNAs (see original paper for the complete list of upregulated and downregulated circRNAs; Peng et al., 2019), and *hsa_circRNA_33287* expression was significantly upregulated during BMP2-induced osteogenic differentiation compared with undifferentiated MSMSCs (Peng et al., 2019). Knockdown of *hsa_circRNA_33287* inhibited osteogenic differentiation as evidenced by decreased accumulation of calcium deposits by alizarin red staining and significant downregulation of osteogenic genes (*ALP*, *RUNX2* and *OSX*) in MSMSCs induced to osteogenic differentiation compared to the control (Peng et al., 2019). Conversely, overexpression of *hsa_circRNA_33287* promoted osteogenic differentiation as evidenced by an increase in the accumulation of calcium deposits and significant upregulation of osteogenic genes (*ALP*, *RUNX2* and *OSX*) in MSMSCs induced to osteogenic differentiation compared to the transfection control (Peng et al., 2019). Mechanistically, *hsa_circRNA_33287* was found to regulate osteogenic differentiation by directly binding and regulating (sponging) *miR-214-3p* in MSMSCs (Peng et al., 2019). Furthermore, *miR-214-3p* was significantly downregulated during BMP2-induced osteogenic differentiation in MSMSCs compared with undifferentiated cells and was shown to directly bind and regulate *RUNX3* (Peng et al., 2019). *In vivo* studies demonstrated that *circRNA_33287* promoted new ectopic bone formation in human MSMSCs subcutaneously transplanted in BALB/c-nu mice (Peng et al., 2019).

The circRNA *hsa_circ_0003489* (*circCDK8*) was investigated in the context of periodontitis, a chronic infection associated with gingival inflammation, periodontal pocket formation and that may lead to alveolar bone loss (Zheng et al., 2021). Periodontal ligament stem cells (PDLSCs) induced to osteogenic differentiation may be an effective treatment for periodontitis-associated bone loss (Zheng et al., 2021) and, more broadly, for bone healing therapeutical strategies. *Hsa_circ_0003489* (*circCDK8*) was found upregulated in human periodontitis tissues compared with control tissues and in PDLSCs treated with CoCl_2_ as an *in vitro* model of hypoxia compared with untreated controls (Zheng et al., 2021). Knockdown of *circCDK8* promoted osteogenic differentiation as evidenced by the significantly increased expression of osteogenic genes (*RUNX2*, *OCN*, *OPN* and *ALP*), increased ALP activity (7 days after osteogenic differentiation) as well as increased accumulation of calcium deposits by alizarin red staining (28 days after osteogenic differentiation) in PDLSCs induced to osteogenic differentiation compared to the control conditions (Zheng et al., 2021).

In the Zhang et al. study (2019), where a microarray analysis was performed in human bone marrow stem cells (BMSCs) to identify differentially expressed circRNAs during osteogenic differentiation (day 7 compared to day 0), the circRNA *hsa_circ_0113689* (*circ-DAB1*) was found to be upregulated (log fold change > 2 and p-value < 0.01) during osteogenic differentiation. As a follow-up from this study, Chia et al. (2020) investigated the expression and role of *circ-DAB1* during the osteogenic differentiation of BMSCs. *Circ-DAB1* was confirmed to be significantly upregulated following 7 days of osteogenic differentiation (compared to day 0) in BMSCs (Chia et al., 2020). Knockdown and overexpression studies demonstrated that *circ-DAB1* regulates the osteogenic differentiation of BMSCs; overexpression of *circ-DAB1* increased the expression of osteogenic genes (*ALP*, *RUNX2*, *OSX*, *OCN* and *COL1A1*) as well as increased ALP activity and the amount of calcium deposits compared to untreated BMSCs, whereas knockdown of *circ-DAB1* reduced the expression of osteogenic genes as well as ALP activity and the amount of calcium deposits compared to untreated BMSCs (Chia et al., 2020). Mechanistically, it was found that *circ-DAB1* regulates (induces) the expression of the *DAB1* gene through the post-transcriptional modulation of *RBPJ* (Chia et al., 2020). Thus, regulation of *RBPJ* expression is mediated by *circ-DAB1* competitively binding to (sponging) *miR-1270* and *miR-944* in BMSCs (Chia et al., 2020).

Finally, *hsa_circ_0003611* (*circLPAR1*) was identified as an exosomal circRNA derived from human primary dental pulp stem cells (DPSCs) undergoing osteogenic differentiation for 5 and 7 days compared to DPSCs’ starvation culture medium (day 0) undifferentiated cells (Xie et al., 2020). Of note, exosomes are membrane-bound extracellular vesicles produced by most eukaryotic cells that carry nucleic acids, proteins, lipids and metabolites (Kalluri and LeBleu, 2020). *Hsa_circ_0003611* (*circLPAR1*) was upregulated in osteogenic-induced DPSC-derived exosomes and increased gradually between osteogenic differentiation days 5 and 7 (Xie et al., 2020). Overexpression of *circLPAR1* promoted the osteogenic differentiation of DPSCs evidenced by increased ALP activity at day 14 of osteogenic differentiation and increased accumulation of calcium deposits by alizarin red staining at day 21 of osteogenic differentiation (Xie et al., 2020). Mechanistically, *circLPAR1* was shown to directly bind and downregulate *hsa-miR-31* to regulate osteogenic differentiation (Xie et al., 2020).

**FIGURE 2:**
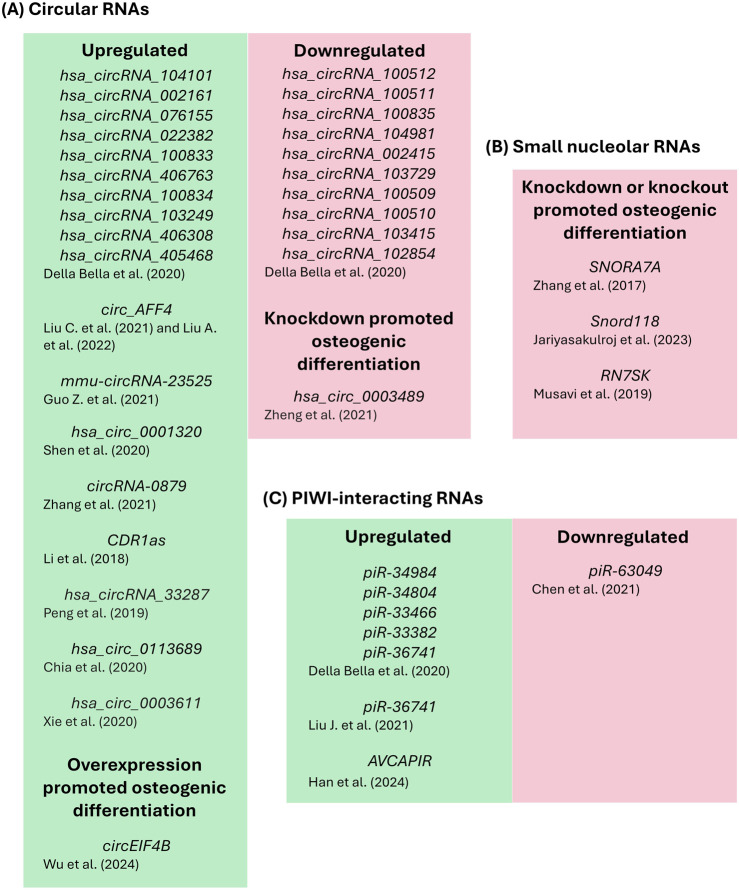
Regulatory and non-coding RNAs modulation and/or biological functions during the osteogenic differentiation of stem, stromal and/or interstitial cells **(A)** Circular RNAs, **(B)** Small nucleolar RNAs and **(C)** PIWI-interacting RNAs.

Altogether, these findings provide novel insights into the complex regulatory system of circRNAs interactions in the cellular microenvironment (summarized in Figure 2A; Table 1) and may enhance the opportunities for novel translational efforts and the development of therapeutical strategies focusing on bone healing and regeneration.

**TABLE 1:** Circular RNAs (circRNAs) biological functions in stem and stromal cells during osteogenic differentiation.

CircRNAs	Model system	Phenotype	References
*hsa_circRNA_104101*	Human primary mesenchymal stromal cells	Cells were induced to osteogenic differentiation for 7 days and *hsa_circRNA_104101* was found to be upregulated.	Della Bella et al. (2020)
*hsa_circRNA_002161*	Human primary mesenchymal stromal cells	Cells were induced to osteogenic differentiation for 7 days and *hsa_circRNA_002161* was found to be upregulated	Della Bella et al. (2020)
*hsa_circRNA_076155*	Human primary mesenchymal stromal cells	Cells were induced to osteogenic differentiation for 7 days and *hsa_circRNA_076155* was found to be upregulated	Della Bella et al. (2020)
*hsa_circRNA_022382*	Human primary mesenchymal stromal cells	Cells were induced to osteogenic differentiation for 7 days and *hsa_circRNA_022382* was found to be upregulated	Della Bella et al. (2020)
*hsa_circRNA_100833*	Human primary mesenchymal stromal cells	Cells were induced to osteogenic differentiation for 7 days and *hsa_circRNA_100833* was found to be upregulated	Della Bella et al. (2020)
*hsa_circRNA_406763*	Human primary mesenchymal stromal cells	Cells were induced to osteogenic differentiation for 7 days and *hsa_circRNA_406763* was found to be upregulated	Della Bella et al. (2020)
*hsa_circRNA_100834*	Human primary mesenchymal stromal cells	Cells were induced to osteogenic differentiation for 7 days and *hsa_circRNA_100834* was found to be upregulated	Della Bella et al. (2020)
*hsa_circRNA_103249*	Human primary mesenchymal stromal cells	Cells were induced to osteogenic differentiation for 7 days and *hsa_circRNA_103249* was found to be upregulated	Della Bella et al. (2020)
*hsa_circRNA_406308*	Human primary mesenchymal stromal cells	Cells were induced to osteogenic differentiation for 7 days and *hsa_circRNA_406308* was found to be upregulated	Della Bella et al. (2020)
*hsa_circRNA_405468*	Human primary mesenchymal stromal cells	Cells were induced to osteogenic differentiation for 7 days and *hsa_circRNA_405468* was found to be upregulated	Della Bella et al. (2020)
*hsa_circRNA_100512*	Human primary mesenchymal stromal cells	Cells were induced to osteogenic differentiation for 7 days and *hsa_circRNA_100512* was found to be downregulated	Della Bella et al. (2020)
*hsa_circRNA_100511*	Human primary mesenchymal stromal cells	Cells were induced to osteogenic differentiation for 7 days and *hsa_circRNA_100511* was found to be downregulated	Della Bella et al. (2020)
*hsa_circRNA_100835*	Human primary mesenchymal stromal cells	Cells were induced to osteogenic differentiation for 7 days and *hsa_circRNA_100835* was found to be downregulated	Della Bella et al. (2020)
*hsa_circRNA_104981*	Human primary mesenchymal stromal cells	Cells were induced to osteogenic differentiation for 7 days and *hsa_circRNA_104981* was found to be downregulated	Della Bella et al. (2020)
*hsa_circRNA_002415*	Human primary mesenchymal stromal cells	Cells were induced to osteogenic differentiation for 7 days and *hsa_circRNA_002415* was found to be downregulated	Della Bella et al. (2020)
*hsa_circRNA_103729*	Human primary mesenchymal stromal cells	Cells were induced to osteogenic differentiation for 7 days and *hsa_circRNA_103729* was found to be downregulated	Della Bella et al. (2020)
*hsa_circRNA_100509*	Human primary mesenchymal stromal cells	Cells were induced to osteogenic differentiation for 7 days and *hsa_circRNA_100509* was found to be downregulated	Della Bella et al. (2020)
*hsa_circRNA_100510*	Human primary mesenchymal stromal cells	Cells were induced to osteogenic differentiation for 7 days and *hsa_circRNA_100510* was found to be downregulated	Della Bella et al. (2020)
*hsa_circRNA_103415*	Human primary mesenchymal stromal cells	Cells were induced to osteogenic differentiation for 7 days and *hsa_circRNA_103415* was found to be downregulated	Della Bella et al. (2020)
*hsa_circRNA_102854*	Human primary mesenchymal stromal cells	Cells were induced to osteogenic differentiation for 7 days and *hsa_circRNA_102854* was found to be downregulated	Della Bella et al. (2020)
*circ_AFF4*	Human bone marrow-derived mesenchymal stem cells	*Circ_AFF4* was upregulated during up to 14 days of osteogenic differentiation. Knockdown of *circ_AFF4* inhibited osteogenic differentiation	Liu C. et al. (2021) Liu A. et al. (2022)
*circEIF4B*	Bone marrow mesenchymal stem cells	*CircEIF4B* overexpression promoted osteogenic differentiation in cells stimulated with phytic acid under a high-glucose microenvironment	Wu et al. (2024)
*mmu-circRNA-23525*	Mouse adipose-derived mesenchymal stem cells	*Mmu-circRNA-23525* was upregulated on osteogenic differentiation days 3, 7 and 14, and *circRNA-23525* over-expression promoted osteogenic differentiation	Guo Z. et al. (2021)
*hsa_circ_0001320* (also named, *circFOXP1*)	Human adipose-derived stem cells	*Hsa_circ_0001320* was upregulated during osteogenic differentiation (days 3, 7 and 14) and *hsa_circ_0001320* overexpression enhanced osteogenic differentiation	Shen et al. (2020)
*CircRNA-0879* (also named, *circRNA-vgll3*)	Adipose-derived stem cells isolated from Sprague Dawley rats	*CircRNA-0879* was upregulated during bone morphogenetic protein 2 (BMP2)-induced osteogenic differentiation (days 2, 7 and 14). Overexpression of *circRNA-0879* regulated BMP2-induced osteogenic differentiation	Zhang et al. (2021)
CircRNA antisense to the cerebellar degeneration-related protein 1 transcript (*CDR1as*)	Human primary periodontal ligament stem cells	*CDR1as* was upregulated following 3, 7 and 14 days of osteogenic differentiation, and knockdown of *CDR1as* inhibited osteogenic differentiation	Li et al. (2018)
*hsa_circRNA_33287*	Primary human maxillary sinus membrane stem cells	*Hsa_circRNA_33287* expression was upregulated during BMP2-induced osteogenic differentiation Moreover, while knockdown of *hsa_circRNA_33287* inhibited osteogenic differentiation, overexpression of *hsa_circRNA_33287* promoted osteogenic differentiation	Peng et al. (2019)
*hsa_circ_0003489* (also named, *circCDK8*)	Human primary periodontal ligament stem cells	*Hsa_circ_0003489* was upregulated in human periodontitis and in human primary periodontal ligament stem cells treated with CoCl_2_ as an *in vitro* model of hypoxia. Knockdown of *hsa_circ_0003489* promoted osteogenic differentiation	Zheng et al. (2021)
*Hsa_circ_0113689* (also named, *circ-DAB1*)	Human bone marrow stem cells	*Hsa_circ_0113689* was upregulated following 7 days of osteogenic differentiation. Overexpression of *hsa_circ_0113689* promoted osteogenic differentiation, while knockdown of *hsa_circ_0113689* inhibited osteogenic differentiation	Chia et al. (2020)
*hsa_circ_0003611* (also named, *circLPAR1*)	Human primary dental pulp stem cells	*Hsa_circ_0003611* was upregulated during osteogenic differentiation days 5 and 7. Overexpression of *hsa_circ_0003611* promoted osteogenic differentiation	Xie et al. (2020)

### 3.2 Small nucleolar RNAs and osteogenic differentiation

SnoRNAs are small (60–300 nucleotides long) non-coding RNAs commonly found in the nucleoli of eukaryotic cells and encoded by introns from both protein coding and non-protein coding genes (Liang et al., 2019; Huang et al., 2022). SnoRNAs are known to play a role in the posttranscriptional chemical modification of ribosomal RNAs and, more recently, have been shown to also participate in the chemical modifications of transfer RNAs, small nuclear RNAs and mRNAs (Bachellerie et al., 2002; Liang et al., 2019; Huang et al., 2022). SnoRNAs are currently classified into three groups depending on the conserved sequence motifs found in the snoRNA molecule: (i) H/ACA box snoRNAs are mainly associated with pseudouridylation, (ii) C/D box snoRNAs are mainly associated with 2′-O-ribose methylation and (iii) small cajal RNAs – located at cajal bodies – that in some cases contain both C/D and H/ACA structures (Bachellerie et al., 2002; Darzacq et al., 2002; Liang et al., 2019; Huang et al., 2022). Due to their effects on RNA chemical modifications, snoRNAs play a role in the folding, stability and protein-interacting properties of their target RNAs (Bratkovič and Rogelj, 2014). The biogenesis and functions of snoRNAs have been previously reviewed elsewhere (Bachellerie et al., 2002; Liang et al., 2019; Bratkovič and Rogelj, 2014; Huang et al., 2022; Bratkovič et al., 2020).

Despite recent advances in our understanding and increasing evidence that snoRNAs play a role in the cellular, tissue and organismal homeostasis and may contribute to the biology of human diseases (McMahon et al., 2015), there are still a lot of unknowns regarding the potential role of snoRNAs in human bone healing and osteogenic differentiation. Of note, despite the nomenclature, the small nucleolar RNA host genes (*SNHGs*) are a family of long non-coding RNAs (and, as such, were not included in this review) that have been shown to play a significant role in human osteogenic differentiation (Xiang et al., 2020; Asila et al., 2021; Zhu et al., 2021; Zhang et al., 2022). The role of *SNHGs* in osteogenic differentiation has been reviewed elsewhere (Tan and Zheng, 2022).

The H/ACA box small nucleolar RNA 7A (*SNORA7A*) was found to be highly expressed in undifferentiated human umbilical cord blood derived mesenchymal stromal cells (uMSCs) and was shown to suppress both osteogenic and adipogenic differentiation of uMSCs (Zhang et al., 2017). In this study, the overexpression of *SNORA7A* significantly inhibited the expression of osteogenic genes as well as the formation of calcium deposits by alizarin red staining in uMSC induced to osteogenic differentiation for 3 weeks, while inhibition of *SNORA7A* induced osteogenic differentiation evidenced by increased expression of osteogenic genes and the formation of calcium deposits by alizarin red staining (Zhang et al., 2017).

More recently, the knockout of *Snord118* – a snoRNA that plays a role as a ribosome biogenesis factor – was shown to cause (i) p53 activation, (ii) increased cell death, (iii) reduced cell proliferation and (iv) early osteogenic differentiation as well as osteoclast loss using Gli1^+^ mouse suture MSCs and/or human induced pluripotent stem cells (iPSC)-derived MSCs as model systems (Jariyasakulroj et al., 2023). The Jariyasakulroj et al. study (2023) investigated the mechanistic role of the *Snord118* in the context of a craniofacial birth defect known as craniosynostosis, which is a result of premature fusion of cranial sutures happening due to the loss of MSCs and an imbalance between bone formation and resorption at the cranial suture site (Jariyasakulroj et al., 2023). Knocking out *Snord118* led – ultimately – to cranial suture growth and craniosynostosis defects (Jariyasakulroj et al., 2023).

*RN7SK* is a conserved snoRNA that plays a role as a scaffold to form complexes with transcription factors, known as the *RN7SK* small nuclear ribonucleoprotein complex, which functions as a regulator of the Polymerase II enzyme activity (Musavi et al., 2019). Expression of *RN7SK* was shown to be downregulated in human MSCs, mouse embryonic fibroblast and mouse AB1 embryonic stem cells in comparison to human fibroblast cells (Abasi et al., 2016). Knockdown of *RN7SK* was demonstrated to induce the osteogenic differentiation potential of human adipose tissue-derived MSCs (AD-MSCs) compared to controls (Musavi et al., 2019). In this study, following osteogenic differentiation for up to 3 weeks, knockdown of *RN7SK* showed significantly higher expression levels of the osteogenic genes *ALP*, *OCN* and *RUNX2* as well as higher levels of alkaline phosphatase activity and calcium deposits investigated by alizarin red staining (Musavi et al., 2019). Altogether, these current results (summarized in Figure 2B; Table 2) demonstrate the role that these snoRNAs (*SNORA7A*, *Snord118* and *RN7SK*) play in regulating osteogenic differentiation and their potential in future translational efforts to modulate the process of bone healing.

**TABLE 2:** Small nucleolar RNAs (snoRNAs) biological functions in stem and stromal cells during osteogenic differentiation.

SnoRNAs	Model system	Phenotype	References
*SNORA7A* Official full name: small nucleolar RNA, H/ACA box 7A	Human umbilical cord blood derived mesenchymal stromal cells	Overexpression of *SNORA7A* inhibited osteogenic differentiation, while inhibition of *SNORA7A* induced osteogenic differentiation	Zhang et al. (2017)
*Snord118* Official full name: small nucleolar RNA, C/D box 118	Gli1^+^ mouse suture mesenchymal stem cells and/or human induced pluripotent stem cells-derived MSCs	Knocking out *Snord118* led to early osteogenic differentiation and osteoclast loss	Jariyasakulroj et al. (2023)
*RN7SK* Official full name: RNA component of 7SK nuclear ribonucleoprotein	Human adipose tissue-derived mesenchymal stem cells	Knockdown of *RN7SK* induced osteogenic differentiation	Musavi et al. (2019)

### 3.3 PIWI-interacting RNAs and osteogenic differentiation

PiRNAs are a class of small non-coding RNAs (24–31 nucleotides long) that are known to associate with the Argonaute family clade of PIWI proteins to form RNA-protein (piRNA-PIWI proteins) complexes, also known as piRNA-induced silencing complexes (Iwasaki et al., 2015). PiRNAs mainly originate from intergenic regions, known as piRNA clusters, and single-stranded RNA-precursors (Iwasaki et al., 2015). PiRNAs and PIWI proteins are highly conserved across evolution and PIWI proteins are mainly expressed in germline cells (gonads) (Iwasaki et al., 2015). As such, the piRNA-PIWI complexes are known to promote transcriptional or post-transcriptional regulation in the activity of transposons (including expression and transposition within the genome), participate in germline development, maintain genomic integrity and stability as well as regulate the degradation of mRNAs (Iwasaki et al., 2015; Yuan et al., 2021). In addition, piRNAs regulate protein-coding genes and may play a role as oncogenes or tumor suppressors by their interaction with different signaling pathways (Iwasaki et al., 2015; Yuan et al., 2021). The biogenesis and functions of piRNAs have been reviewed elsewhere (Iwasaki et al., 2015; Yuan et al., 2021; Wang et al., 2023; Haase, 2022).

In a study using human primary mesenchymal stromal cells induced to osteogenic differentiation for 7 days, RNA sequencing identified 8 upregulated piRNAs and 46 downregulated piRNAs compared to the control as early markers of osteogenic lineage differentiation commitment (Della Bella et al., 2020). In this study, 5 piRNAs were found upregulated with a fold change ≥ 2 as follows: *piR-34984* (piRBase ID: *piR-hsa-27208*), *piR-34804* (piRBase ID: *piR-hsa-26940*), *piR-33466* (piRBase ID: *piR-hsa-23617*), *piR-33382* (piRBase ID: *piR-hsa-23533*) and *piR-36741* (piRBase ID: *piR-hsa-28875*) (Della Bella et al., 2020). Moreover, downregulated piRNAs with a fold change range between 0.1–0.6 were reported [see original paper for a complete list of upregulated and downregulated piRNAs (Della Bella et al., 2020)]. Unfortunately, while some independent validation of the RNA sequencing findings was performed by quantitative PCR, no upregulated or downregulated piRNAs were shown to be validated during osteogenic differentiation in the study (Della Bella et al., 2020). Of interest, however, the study generated a significant amount of RNA sequencing data that is currently publicly available in human primary mesenchymal stromal cells during an early stage of osteogenic (and chondrogenic) commitment (Della Bella et al., 2020).

As an independent follow-up of the Della Bella et al. study (2020), *piR-36741* was reported to be significantly upregulated following 7 and 14 days of osteogenic differentiation in primary human BM-MSCs compared to day 0 (Liu J. et al., 2021). Moreover, inhibition of *piR-36741* expression significantly delayed osteogenic differentiation in primary human BM-MSCs as evidenced by the lower expression of osteogenic genes and proteins (RUNX2, collagen type I alpha 1 chain, osteocalcin and osteopontin), lower levels of alkaline phosphatase activity as well as lower levels of calcium deposits in *piR-36741* silenced cells compared to controls (Liu J. et al., 2021). Mechanistically, it was found that *piR-36741* binds to the enzyme methyltransferase 3 (METTL3) and regulates the activity of METTL3 to promote N6-methyladenosine (m6A) methylation of the bone morphogenetic protein 2 (*BMP2*) mRNA (Liu J. et al., 2021). Of interest for future translational applications in bone formation and healing, the administration of mimic-piR-36741 in ovariectomy-induced osteoporosis mice demonstrated significant improvements in bone structure, bone trabecula thickness, bone density, bone strength and bone elasticity coefficient compared to the control group (Liu J. et al., 2021).

*PiR-63049* was investigated in the context of bone remodeling (Chen et al., 2021), the lifelong process where mature bone tissue is removed from the skeleton (a process known as bone resorption and mainly mediated by osteoclasts) and new bone tissue is formed, a process known as bone formation and mainly mediated by osteoblasts. The study from Chen et al. (2021) identified *piR-63049* as an osteoporosis-related piRNA in the bone marrow stromal cells from ovariectomized rats. Of note, the RNA sequencing investigation identified a total of 13 upregulated and 15 downregulated piRNAs (fold change ≥ 2 and *p*-value < 0.05) in the bone marrow stromal cells from ovariectomized rats [see original paper for the complete list of upregulated and downregulated piRNAs (Chen et al., 2021)]. Chen et al. also demonstrated that *piR-63049* is significantly downregulated in bone marrow stromal cells after 7 and 14 days of osteogenic induction (Chen et al., 2021). Mechanistically, overexpression of *piR-63049* inhibited osteogenic differentiation of bone marrow stromal cells, while knocking down the expression of *piR-63049* promoted the osteogenic differentiation of bone marrow stromal cells through the Wnt/β-catenin signaling pathway (Chen et al., 2021). Moreover, using a *piR-63049*-antagonist attenuated bone loss and promoted bone formation in the ovariectomized rat model of osteoporosis (Chen et al., 2021). Of interest, the expression of *piR-63049* (for humans, the homologous is *piR-hsa-174699*) was significantly increased in the bone tissues and plasma of postmenopausal osteoporotic patients (Chen et al., 2021). Altogether, these results suggest that targeting *piR-63049* may be a potential approach to modulate bone formation for clinical and translational applications.

Recently, a novel aortic valve calcification-associated PIWI-interacting RNA (*piR-hsa-25624*, renamed *AVCAPIR*) was identified in the context of increasing valvular calcification and promoting the progression of calcific aortic valve disease (Han et al., 2024). *AVCAPIR* was found to be significantly upregulated in aortic valve calcification, and deletion of *AVCAPIR* ameliorated aortic valve calcification *in vivo* as evidenced by reduced thickness and calcium deposition in the aortic valve leaflets as well as downregulation of osteogenic markers in the aortic valves (Han et al., 2024). Similar results were observed following 14 days of osteogenic differentiation of human primary valvular interstitial cells (Han et al., 2024). Mechanistically, it was found that *AVCAPIR* interacts with the fat mass and obesity-associated protein and blocks its *N* ^6^-methyladenosine demethylase activity of CD36 mRNA transcripts (Han et al., 2024). As a result, CD36 stabilizes and binds to proprotein convertase subtilisin/kexin type 9 (PCSK9), which ultimately accelerates the development and progression of aortic valve calcification (Han et al., 2024). Additional upregulated and downregulated piRNAs (160 upregulated and 144 downregulated piRNAs with a fold change ≥ 2 and adjusted *p*-value < 0.05) were identified from aortic valve samples from patients with and without calcific aortic valve disease (see original paper; Han et al., 2024).

Despite its novelty, a landscape for piRNAs’ role in osteogenic differentiation and bone formation (summarized in Figure 2C; Table 3) is starting to evolve and demonstrates a significant potential for future clinical and translational bone healing applications.

**TABLE 3:** PIWI-interacting RNAs (piRNAs) biological functions in stem, stromal and interstitial cells during osteogenic differentiation.

PiRNAs	Model system	Phenotype	References
*piR-34984* piRBase ID: *piR-hsa-27208*	Human primary mesenchymal stromal cells	Cells were induced to osteogenic differentiation for 7 days and *piR-34984* was upregulated	Della Bella et al. (2020)
*piR-34804* piRBase ID: *piR-hsa-26940*	Human primary mesenchymal stromal cells	Cells were induced to osteogenic differentiation for 7 days and *piR-34804* was upregulated	Della Bella et al. (2020)
*piR-33466* piRBase ID: *piR-hsa-23617*	Human primary mesenchymal stromal cells	Cells were induced to osteogenic differentiation for 7 days and *piR-33466* was upregulated	Della Bella et al. (2020)
*piR-33382* piRBase ID: *piR-hsa-23533*	Human primary mesenchymal stromal cells	Cells were induced to osteogenic differentiation for 7 days and *piR-33382* was upregulated	Della Bella et al. (2020)
*piR-36741* piRBase ID: *piR-hsa-28875*	Human primary mesenchymal stromal cells	Cells were induced to osteogenic differentiation for 7 days and *piR-36741* was upregulated	Della Bella et al. (2020)
*piR-36741* piRBase ID: *piR-hsa-28875*	Human primary bone-marrow mesenchymal stem cells	*PiR-36741* was upregulated following 7 and 14 days of osteogenic differentiation. Inhibition of *piR-36741* expression delayed osteogenic differentiation. Treatment of ovariectomy-induced osteoporosis mice with a mimic-piR-36741 improved bone structure, bone trabecula thickness and bone density, among others	Liu J. et al. (2021)
*piR-63049* piRBase ID: *piR-hsa-174699*	Bone marrow stromal cells from ovariectomized rats	*PiR-63049* is downregulated after 7 and 14 days of osteogenic differentiation. In addition, overexpression of *piR-63049* inhibited osteogenic differentiation, while knocking down *piR-63049* promoted osteogenic differentiation. Using a *piR-63049*-antagonist attenuated bone loss and promoted bone formation *in vivo*	Chen et al. (2021)
*AVCAPIR* piRBase ID: *piR-hsa-25624*	Human valvular interstitial cells and high-cholesterol diet-fed ApoE^−/−^ mice with *AVCAPIR* knockout	*AVCAPIR* is upregulated during osteogenic differentiation. *AVCAPIR* was also found to be upregulated in aortic valve calcification, and deletion of *AVCAPIR* ameliorated aortic valve calcification *in vivo*	Han et al. (2024)

## 4 Discussion

Regulatory and non-coding RNAs play essential roles in multiple cellular processes, including osteogenic differentiation and bone healing. A better understanding of the molecular mechanisms underlying osteogenic commitment and differentiation will allow us to develop effective targeted approaches toward bone healing and tissue regeneration.

Multiple regulatory circRNAs have been found to play a role in the osteogenic differentiation of stem and stromal cells (summarized in Figure 2A; Table 1). CircRNAs are known to participate in a diverse range of cellular processes, including (i) acting as microRNAs sponges to modulate (upregulate) the expression of their mRNA targets (Hansen et al., 2013), (ii) regulating transcription and translation (Li et al., 2015; Wu et al., 2019) and (iii) promoting and/or facilitating interactions between proteins (Fang et al., 2018; Wu et al., 2019). Multiple studies summarized here have focused their mechanistic efforts to identify the downstream targets from each respective circRNA, including the targeted microRNA that is directly regulated (sponged) by the circRNA as well as the downstream mRNA that is modulated as a result of the absence of the sponged microRNA. While this is an important layer of regulation, it is only one layer of regulation and does not include any other potential role(s) associated with these circRNAs during the osteogenic differentiation process. As such, the identification of additional relevant roles for circRNAs in the osteogenic differentiation of stem and stromal cells remains a fundamental challenge to be addressed.

In comparison to circRNAs, both snoRNAs and piRNAs have been less studied during the processes of osteogenic commitment and differentiation of stem, stromal and interstitial cells (summarized in Figures 2B, C; Tables 2, 3, respectively). SnoRNAs are known to play a role in the post-transcriptional chemical modification of ribosomal RNAs, transfer RNAs, small nuclear RNAs and mRNAs (Bachellerie et al., 2002; Liang et al., 2019; Huang et al., 2022). PiRNAs are known to associate with the Argonaute family clade of PIWI proteins to form piRNA-induced silencing complexes (Iwasaki et al., 2015). The piRNA-PIWI complexes are known to (i) promote transcriptional or post-transcriptional regulation in the activity of transposons (including expression and transposition within the genome), (ii) participate in germline development, (iii) maintain genomic integrity and stability as well as (iv) regulate the degradation of mRNAs (Iwasaki et al., 2015; Yuan et al., 2021). The functional roles of snoRNAs and piRNAs during the osteogenic differentiation process are just starting to be identified and elucidated and will likely be investigated in the future for the development of targeted therapeutic strategies for bone fractures that focus on bone healing and tissue regeneration.

Recent advances in the field of RNA biology have uncovered a role for regulatory and non-coding RNAs as disease biomarkers and as novel druggable targets for therapeutical interventions. In general, multiple RNA-based treatments have been approved and/or are part of preclinical studies and clinical trials (reviewed elsewhere; Zhu et al., 2022). Selectively and specifically targeting these regulatory and non-coding RNAs offers new opportunities in drug development, including novel (i) mechanisms of action and (ii) druggable targets to be explored. In summary, the potential to target regulatory and non-coding RNAs selectively and specifically might revolutionize the field of orthopedics, including – but not limited to – the treatment of post-traumatic injuries, bone fractures, bone healing and tissue regeneration. However, more research is still needed to elucidate the molecular mechanisms of circRNAs, snoRNAs and piRNAs during osteogenic differentiation and more completely understand the systemic and long-term effects of targeting these regulatory and non-coding RNAs in humans. Finally, it is critical to elucidate and validate their potential clinical benefit, including efficacy, safety and potential side effects for the treatment of bone fractures in humans. Overall, the regulatory and non-coding RNAs circRNAs, snoRNAs and piRNAs may be promising therapeutic targets in bone regenerative medicine.
